# Multimodality Non-Invasive Imaging Approach in Acute Coronary Syndrome: Diagnostic and Prognostic Assessment

**DOI:** 10.1007/s11886-025-02286-9

**Published:** 2025-11-27

**Authors:** Daniele Cavallo, Luca Bergamaschi, Francesco Angeli, Matteo Armillotta, Ornella Di Iuorio, Khrystyna Ryabenko, Claudio Asta, Nicole Suma, Mariachiara Ciarlantini, Damiano Fedele, Lisa Canton, Sara Amicone, Rebecca Belà, Leonardo Luca Bavuso, Jessica Salerno, Marcello Casuso Alvarez, Marco Basile, Angelo Maida, Tommaso Manaresi, Nicolò Vasumini, Michele Di Leo, Domenico Tuttolomondo, Roberto Carletti, Gianni Dall’Ara, Elisa Gardini, Maria De Vita, Nicola Gaibazzi, Marco Guglielmo, Anna Giulia Pavon, Giuseppe Ciliberti, Angelo Squeri, Giancarlo Facchini, Gianluca Pontone, Carmine Pizzi

**Affiliations:** 1 Department of Medical and Surgical Sciences—DIMEC—Alma Mater Studiorum, University of Bologna, Bologna, 40138 Italy; 2 Cardiology Unit, Cardiac Thoracic and Vascular Department, IRCCS Azienda Ospedaliera-Universitaria di Bologna, Bologna, 40138 Italy; 3 Cardiovascular Division, Morgagni-Pierantoni University Hospital, Forlì, Italy; 4 Department of Cardiology, Parma University Hospital, Parma, 43126 Italy; 5 Department of Cardiology, Division of Heart and Lungs, Utrecht University Medical Center, Utrecht, The Netherlands; 6 Department of Cardiology, Haga Teaching Hospital, The Hague, The Netherlands; 7 Department of Cardiology, Cardiocentro Ticino Institute, Ente Ospedaliero Cantonale, Via Tesserete, 48, Lugano, 6900 Switzerland; 8 Cardiology and Arrhythmology Clinic, Marche Polytechnic University, Azienda Ospedaliero Universitaria delle Marche, Ancona, Italy; 9 Interventional Cardiology Unit, Maria Cecilia Hospital, GVM Care and Research, Cotignola, RA Italy; 10 Department of Perioperative Cardiology and Cardiovascular Imaging, Centro Cardiologico Monzino IRCCS, Milan, Italy; 11 Diagnostic and Interventional Radiology Unit, IRCCS Istituto Ortopedico Rizzoli, Bologna, Italy; 12 Department of Biomedical, Surgical and Dental Sciences, University of Milan, Milan, Italy

**Keywords:** Imaging, Multimodality, TTE, CCTA, CMR, ACS

## Abstract

**Purpose of review:**

The diagnostic, therapeutic, and prognostic management of patients with suspected acute coronary syndrome (ACS) is a major challenge for clinicians in both emergency and outpatient settings. While clear-cut cases of acute myocardial infarction typically require immediate invasive coronary angiography (ICA), more nuanced and complex presentations require careful selection of the most appropriate diagnostic tests to determine the underlying cause of symptoms. This narrative review aims to delineate specific scenarios in which non-invasive multimodal imaging techniques—such as transthoracic echocardiography (TTE), coronary computed tomography angiography (CCTA), cardiac magnetic resonance (CMR), and nuclear imaging—are appropriate and optimal in the setting of ACS.

**Recent findings:**

In the initial assessment of a patient with suspected ACS, TTE is essential to identify regional wall motion abnormalities (RWMA) with a typical “coronary pattern”. In recent years, the use of speckle tracking echocardiography has been shown to increase diagnostic sensitivity in this setting, particularly in patients without overt RWMA. Stress echocardiography also holds diagnostic value in specific low-risk ACS settings. Moreover, in this patient population, CCTA has demonstrated a very high negative predictive value (NPV) across multiple trials, effectively reducing the number of unnecessary ICA. Recently, this technique has been enhanced by the ability to perform qualitative analysis of atherosclerotic plaque, allowing the identification of high-risk features associated with instability and rupture, and thus with ACS. Finally, CMR enables myocardial tissue characterization, which is essential in the diagnostic work-up of myocardial infarction with non-obstructive coronary arteries (MINOCA) and also serves as an effective gatekeeper in suspected non-ST elevation myocardial infarction (NSTEMI) through the exclusion of mimickers such as myocarditis, thereby reducing the number of useless ICA. Moreover, CMR is supported by substantial evidence regarding its prognostic value in ACS patients. When available, myocardial perfusion imaging, using single photon emission tomography or positron emission tomography, has a valuable role in patients with suspected ACS and non-diagnostic ECG and biomarkers; in fact, it can detect inducible ischemia and prior infarction with a high NPV supporting safe discharge and reducing unnecessary admissions.

**Summary:**

We aim to point out the role of non-invasive multimodal imaging in patients with confirmed or suspected ACS. By analyzing the available evidence and current guidelines, it’s clear that these imaging techniques are especially useful in cases of low pre-test ACS probability, low-risk NSTEMI, in ruling out alternative diagnoses, and in specific diagnostic work-up such as MINOCA. In clinical practice, our goal is to provide practical recommendations for the clinicians on when and how to apply non-invasive imaging to reduce the number of ICA in order to minimize redundant, costly, and invasive diagnostic procedures that carry an inherent risk of complications.

**Graphical Abstract:**

Non-invasive imaging in the diagnostic pathway of suspected NSTE-ACS. * This refers to patients with acute myocardial injury but low levels of cTn, low pre-test probability of CAD (no history of previous MI, known CAD or revascularizations) or with possible alternative causes of myocardial injury.† Depending on center expertise and availability, either stress echocardiography, rest/stress SPECT/PET, stress CT perfusion and stress CMR can also be used.
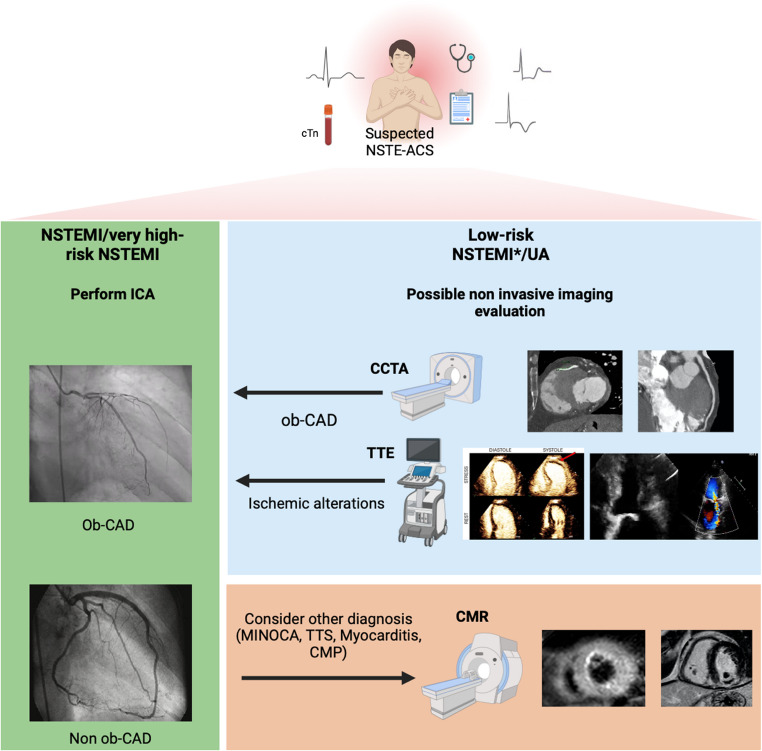

## Introduction

Acute coronary syndrome (ACS) includes ST-elevation myocardial infarction (STEMI), non-ST-elevation myocardial infarction (NSTEMI), and unstable angina (UA) occurring without myocardial damage. In this context, invasive coronary angiography (ICA) plays an important diagnostic and therapeutic role. However, it carries a potential risks of adverse events [1]. Recently advances in non-invasive imaging technologies have paved the way for their potential use in ACS setting.

Transthoracic echocardiography (TTE) remains the first line imaging modality providing immediate information about myocardial contractility alterations and detecting potential mechanical complications of acute myocardial infarction (AMI). Coronary computed tomography angiography (CCTA) provides a rapid, non-invasive visualization of the coronary anatomy. CT scan also helps to rule out non-coronary causes of myocardial damage such as myocarditis and extracardiac causes such as pulmonary embolism (PE) and acute aortic syndromes, too. Finally, cardiac magnetic resonance (CMR) is the gold standard for cardiac function evaluation and tissue characterization of myocardial damage.

Current guidelines provide limited clarity on the appropriate use and timing of these diagnostic tools. This paper aims to provide a comprehensive systematic review of non-invasive multimodal imaging modalities for the diagnostic assessment and prognostic stratification of patients with suspected ACS.

## Transthoracic Echocardiography

### Diagnostic Phase

Currently, TTE is the primary non-invasive imaging modality for ACS due to its availability, versatility, and relative safety. It is particularly useful in patients with acute chest pain or dyspnea of unclear origin, as it might reveal signs of ischemia or prior AMI [1]. TTE helps to correlate specific coronary vessels with regional wall motion abnormalities (RWMA), creating a “coronary pattern” that can be used alongside electrocardiogram (ECG) findings to help to identify the culprit coronary artery. In addition, the Wall Motion Score Index (WMSI) provides a semi-quantitative assessment, although it faces challenges such as limited reproducibility and operator dependence [2] (Table 1).

**Table 1:** Selected studies on the diagnostic and prognostic role of TTE in the ACS setting. TTE, transthoracic echocardiography; CAD, coronary artery disease; NSTE-ACS, Non ST elevation acute coronary syndrome; NSTEMI, Non-ST elevation myocardial infarction; cTn, cardiac troponin; ICA, invasive coronary angiography; pPCI, primary percutaneous coronary intervention; STEMI, ST-elevation myocardial infarction; LVEF, left ventricular ejection fraction; STE, speckle tracking echocardiography; WMSI, wall motion score index; GLS, global longitudinal strain; ESVI, end-systolic volume index; SE, stress echocardiography; S-MCE, stress-myocardial contrast echocardiography; MACE, major adverse cardiovascular events; RCT, randomized-controlled trial

IMAGING METHOD	STUDY	STUDY DESIGN	POPULATION	ENDPOINT	RESULTS
TTE	Dahlslett et al. [3] 2014	Observational	64 patients with suspected NSTE-ACS without known CAD, inconclusive ECG findings, and normal cTn. All patients underwent ICA	Correlation between STE and CAD	GLS was superior to LVEF, WMSI and GRACE score in distinguishing patients with and without significant CAD
	Grenne et al. [4] 2010	Observational	111 patients with suspected NSTE-ACS (67 NSTEMI diagnosis)	Comparison between STE and conventional TTE parameters	Territorial circumferential strain enables very early identification of acute coronary occlusions
	Boe et al. [5] 2015	Observational	126 patients with suspected NSTE-ACS scheduled for ICA	Ability of myocardial work index to identify acute coronary occlusion	Myocardial work index was superior to GLS and LVEF to identify acute coronary occlusion
	Chelliah et al. [8] 2010	Observational	547 patients with new onset chest pain and without CAD history	Prognostic value of SE	SE had independent and incremental prognostic value for predicting of MACE above clinical, ECG, and stress ECG data
	HORIZONS-AMI trial [11] 2014	RCT	2648 patients admitted for STEMI underwent pPCI	Prognostic value of LVEF	MACE markedly increased in group with LVEF < 40%
	Munk et al. [12] 2012	Observational	576 patients admitted for STEMI < 24 h after pPCI	Prognostic value of GLS and comparison with LVEF, WMSI and ESVI	GLS and WMSI were comparable and both superior for early risk assessment compared with LVEF and ESVI.
	Ersbøll et al. [13] 2013	Observational	849 patients admitted for AMI and preserved LVEF (> 40%)	Prognostic value of GLS	GLS had independent prognostic value, specifically GLS > −14% was significantly associated with cardiovascular death and heart failure hospitalization
	Haugaa et al. [14] 2013	Observational	569 patients > 40 days after AMI	Arrhythmic events prediction with STE	GLS and mechanical dispersion predicted arrhythmic events (sustained ventricular tachycardia and sudden cardiac death) independently of LVEF
	CROSS-AMI trial [18] 2019	RCT	306 patients admitted for STEMI and multivessel CAD	Prognostic comparison between complete angiographically and SE-guided revascularization	SE–guided revascularization was similar to complete angiographically guided revascularization and reduced elective revascularization before hospital discharge
	Gaibazzi et al. [19] 2011	Observational	545 patients with suspected NSTE-ACS but non-diagnostic ECG and normal cTn values	Prognostic value of S-MCE	S-MCE provided independent information for predicting MACE beyond SE only

Speckle tracking echocardiography (STE) improves the diagnostic accuracy of ischemic patterns, particularly in differentiating between ischemic and non-ischemic conditions. In non-ST elevation ACS (NSTE-ACS) cases with equivocal ECG results and normal cardiac troponin (cTn) levels, normal global longitudinal strain (GLS) has a high negative predictive value (NPV) for significant coronary artery disease (CAD) [3]. Segmental contractility analysis using myocardial regional strain might help to detect earlier acute coronary occlusions in patients with NSTE-ACS, with circumferential strain showing excellent sensitivity and specificity for this purpose [4]. In this setting, myocardial work, defined as a parameter to evaluate the efficiency of the myocardial muscle contraction (GLS x blood pressure), has shown superiority over GLS and left ventricular ejection fraction (LVEF) [5].

In complex cases, especially in the intensive care units, myocardial contrast echocardiography (MCE) can further clarify RWMA detection and improve interobserver agreement compared to unenhanced echocardiography [6]. In addition, MCE assessment of myocardial perfusion defects by MCE complements RWMA analysis for accurate diagnosis of an ACS [7]. However, the routine use of MCE is limited by the need for specialized training. Clinically, TTE can also identify alternative conditions that mimic ACS, such as acute aortic dissection (especially type A), significant valve disease (e.g., aortic valve stenosis), PE, and Takotsubo cardiomyopathy.

**Fig. 1:**
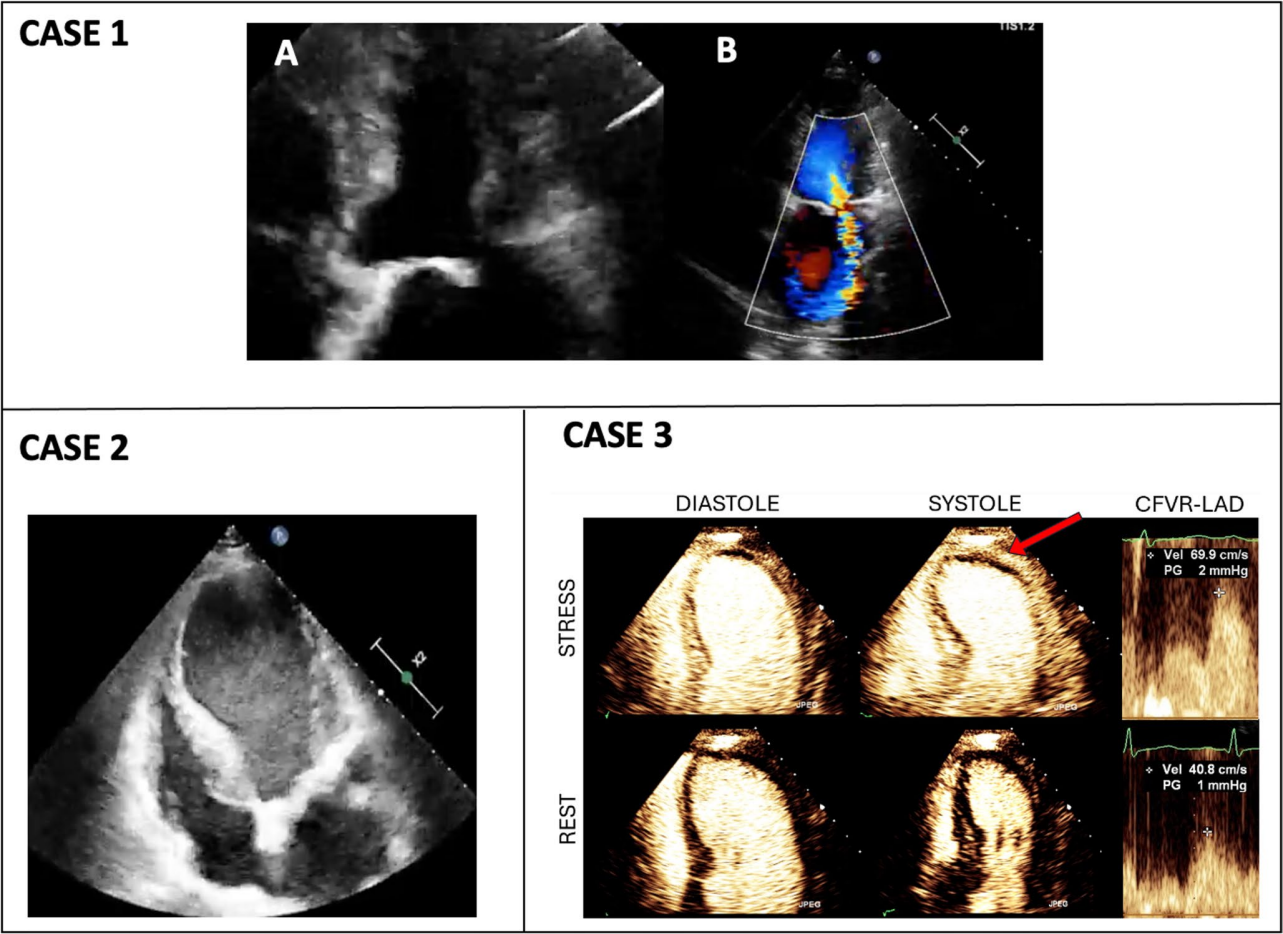
Echocardiographic evaluation in ACS. *Case 1*: Late-presenter STEMI with thrombotic occlusion of the first diagonal and intermediate branches, causing partial rupture of the antero-lateral papillary muscle and severe mitral regurgitation (1A, 1B). *Case 2*: Aneurysmal LV apex following anterior STEMI with 'smoke' effect. *Case 3*: Suspected UA patient underwent stress MCE post-ED discharge, showing normal wall motion at rest. After dipyridamole infusion, systolic akinesia of the LV apex (arrow) with perfusion defect and reduced CFVR-LAD of 1.7. Abbreviations:MCE, myocardial contrast echocardiography; ED, emergency department; LV, left ventricular; CFVR, coronary flow velocity reserve**, **and as in Figure 1

It is essential to rule out mechanical complications associated with late-onset STEMI, such as ventricular wall rupture and atrioventricular valve regurgitations secondary to papillary muscle rupture **(**Fig. 1, case 1 and 2). Rapid recognition of these complications is crucial for prompt therapy.

Stress echocardiography (SE) is used to identify inducible ischemia, especially in chronic settings, where the new ABCDE, by evaluating five different functional reserves and phenotypes, allows a comprehensive risk stratification beyond the simple coronary artery stenosis. However, it may be useful in some equivocal cases of NSTE-ACS where chest pain occurs in the absence of ischemic changes on ECG and normal high-sensitivity cTn (hs-cTn) levels. It has also been shown that patients with new-onset angina and negative cardiovascular history could benefit from SE rather than stress ECG [8]. Exercise echocardiography is preferred, although pharmacologic options such as dobutamine and vasodilator testing are available for patients who are unable to exercise. However, routine SE is contraindicated in AMI within three days and ongoing UA [9].

Finally, the use of stress MCE has been shown to be superior over Thrombolysis in Myocardial Infarction (TIMI) risk score and stress ECG in the assessment of suspected NSTE-ACS with non-diagnostic ECG and negative cTn [10] **(**Fig. 1, case 3).

### Prognostic Role

Left ventricular (LV) dysfunction has the key prognostic value in patients with ACS and should be assessed before hospital discharge [1]. This recommendation from the latest ESC guidelines is derived from several randomized controlled trials (RCTs), such as Horizons-AMI trial which showed a significantly higher incidence of major adverse cardiovascular events (MACE) in ACS patients with impaired LVEF [11].

Other echocardiographic characteristics can be assessed to predict outcomes in ACS patients, such as GLS or WMSI. GLS and WMSI have been shown to be superior to LVEF and end-systolic volume index for early risk stratification of STEMI patients after primary percutaneous coronary intervention (pPCI) [12]. In patients with preserved LVEF after AMI, an impaired GLS is associated with a higher risk of heart failure and all-cause mortality [13]. The prognostic value of this tool also extends to the arrhythmic complications of AMI, in fact GLS and mechanical dispersion have been shown to predict sustained ventricular tachycardia and sudden cardiac death after AMI, independent of LVEF [14]. Myocardial work can also accurately stratify NSTE-ACS patients after PCI, providing a new non-invasive method for clinical postoperative assessment of myocardial function [15, 16].

SE is most useful for the prognostic stratification of patients presenting to the emergency department (ED) with chest pain of suspected ischemic origin, in the absence of ECG changes and cTn elevation. In these cases, SE or stress MCE performed within 5 days from the index event has demonstrated that inducible ischemia accurately predicts the 1-year incidence of ACS (11.3% for positive results vs. 0.8% for negative results) [17]. SE is also useful in guiding complete revascularization in patients hospitalized for STEMI and multivessel CAD. The results of the CROSS-AMI trial showed that a SE–guided revascularization strategy is safer and non-inferior to an angiography-guided strategy in the prediction of MACE [18]. Furthermore, stress MCE adds prognostic information to SE alone in suspected NSTE-ACS [19].

Microcirculatory dysfunction is a well-known complication of STEMI and coronary flow velocity reserve (CFVR) is a useful tool for predicting recovery of LV function after revascularization in patients with AMI [20], but further evidence is needed.

## Coronary Computed Tomography Angiography

### Diagnostic Phase

Currently, CCTA offers a straightforward anatomical assessment of the coronary tree while providing detailed insights into plaque burden, qualitative assessment of atherosclerotic plaques, identification of high-risk features, inflammation, wall motion analysis, myocardial scar and fibrosis, as well as the percentage of myocardium at risk and risk scores such as the Leaman score [21]. In addition, CCTA-derived functional assessment techniques, such as fractional flow reserve-CT and CT perfusion enhance the diagnostic specificity of this modality [22]. While these techniques are validated for chronic coronary syndrome, they have not yet been confirmed for use in ACS.

**Fig. 2:**
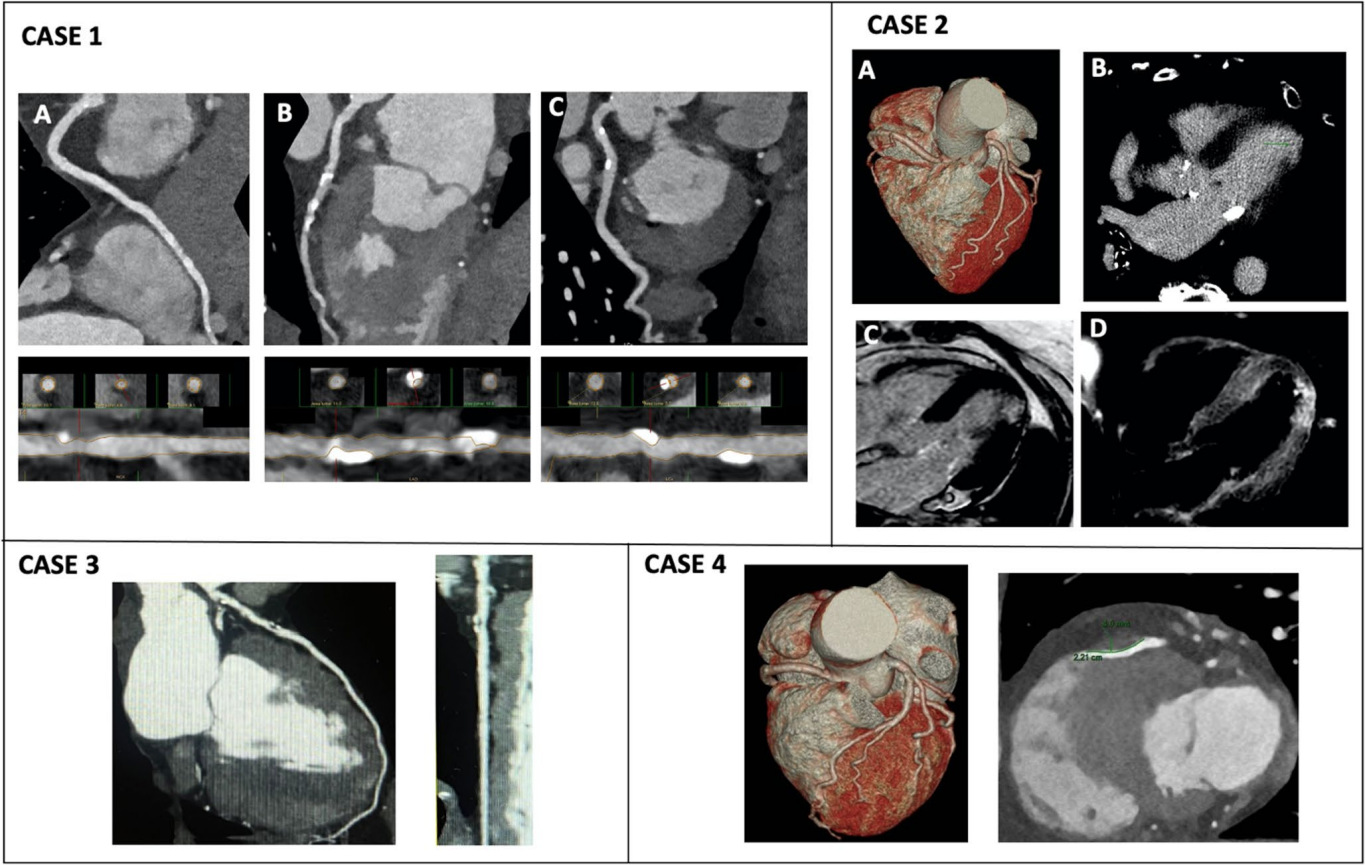
CCTA evaluation in ACS. *Case 1:* A patient with suspected NSTE-ACS underwent CCTA, revealing severe stenosis due to significant calcifications in the mid-proximal LAD (1B), confirmed by ICA. The RCA (1A) and circumflex branch (1C) showed non-critical fibro-calcific plaques. *Case 2*: A patient with chest pain and acute myocardial injury had a CT scan for triple rule-out, showing no obstructive CAD (2A) and excluding PE and acute aortic syndrome. CCTA revealed a focal ischemic area in the apical-lateral segment (2B), confirmed by CMR with subendocardial-intramyocardial LGE (2C) and T2 hyperintensity indicating edema (2D), extending to the apical-septal segment. *Case 3:* A patient with UA had CCTA showing a 75% stenosis with high-risk atheromatous plaque (napkin-ring sign, positive remodeling). *Case 4:* A patient with recurrent chest pain had ECG ischemic changes and mild hs-cTn elevation, leading to ICA, which showed no significant coronary lesions (4A). However, CCTA revealed an intramyocardial course of the mid-segment of the LAD (depth 4 mm, length 2.2 cm). Abbreviations:LAD, left anterior descending artery; RCA, right coronary artery; CT, computed tomography; CAD, coronary artery disease; PE, pulmonary embolism; LGE, late-gadolinium enhancement, and as inFigure 1

In ED, CCTA can simultaneously exclude obstructive CAD (ob-CAD) and other causes of acute chest pain, such as PE and aortic dissection, using a “triple rule-out” protocol **(**Fig. 2, case 2). It is also effective in diagnosis of congenital coronary artery anomalies, myocardial bridges and spontaneous coronary artery dissection; though, regarding the latter, the diagnostic gold standard remains ICA with optical coherence tomography (OCT) or intravascular ultrasound (IVUS) especially in case of involvement of distal coronary arteries or side branches with a vessel caliber < 2.5 mm, usually not well visualized on CCTA [23]. However, certain limitations affect the applicability of CCTA, including severe calcifications, a history of previous revascularization, irregular/elevated heart rate, and the unavailability of 24-hour service in many locations despite these are considered relative contraindications thanks to the introduction of last generation scanners. In addition, CT scan allows myocardial tissue characterization through late iodine enhancement and extracellular volume, which is useful for the diagnosis of myocarditis in patients with acute chest pain, as shown by Palmisano et al. [24]

Numerous studies have evaluated CCTA in patients presenting to the ED with suspected NSTE-ACS. In this context, CCTA is recommended for low to intermediate risk patients (hemodynamically stable, normal initial cTn levels, non-ischemic ECG, and no history of CAD), and has demonstrated high sensitivity and NPV to rule out ob-CAD [25].

In the early 2000 s, several RCTs compared CCTA with standard of care (SOC) in low to intermediate risk patients with acute chest pain **(**Table 2**).** These studies showed that a CCTA-based strategy allowed safer and faster discharge for patients without ob-CAD [26]. For example, the ROMICAT II-trial [27] found that patients who underwent early CCTA had a shorter mean hospital length of stay, a faster diagnosis and a higher rate of direct discharge from the ED. More diagnostic functional tests were performed in the CCTA group, probably due to the identification of suspicious non-critical CAD.

**Table 2:** Selected studies on the diagnostic and prognostic role of CCTA in the ACS setting. ED, emergency department; CCTA, coronary computed tomography angiography; SOC, standard of care; MPI, myocardial perfusion imaging; hs-cTn, high sensitivity cardiac troponin; PCAT, pericoronary adipose tissue; ob-CAD, obstructive coronary artery disease; HRP, high risk plaque; N/PPV, negative/positive predictive value

IMAGING METHOD	STUDY	STUDY DESIGN	POPULATION	ENDPOINT	RESULTS
CCTA	ROMICAT trial [25] 2009	Trial	368 patients with suspected NSTE-ACS in ED with normal cTn and nonischemic ECG	Correlation between CCTA results and ACS during index hospitalization and MACE during 6-month follow-up	CCTA showed elevated sensitivity and NPV (100% of patients with no CAD had no ACS and subsequent MACE) but limited PPV
	ACRIN-PA trial [26] 2012	RCT	1,370 patients (CCTA vs. SOC) with suspected NSTE-ACS in ED with normal ECG and TIMI risk score 0–2	AMI and cardiac death during the first 30 days after ED presentation in patients without ob-CAD at CCTA	CCTA showed safety to quick discharge patients from ED
	ROMICAT II trial [27] 2012	RCT	1,000 patients (CCTA vs. SOC) with suspected NSTE-ACS ED with nonischemic ECG and normal cTn	Length of stay in the hospital, rates of discharge from ED, MACE and costs at 28 days	CCTA reduced length of hospital stay of 7.6 h, led to a quicker diagnosis and higher rate of direct discharge from ED (47% vs. 12%), no differences in MACE or costs
	CT-STAT trial [28] 2011	RCT	749 patients (CCTA vs. MPI) with suspected NSTE-ACS in ED with nonischemic ECG and normal cardiac biomarkers	Time to diagnosis, ED costs and MACE over 6 months	CCTA showed 54% reduction in time to diagnosis and a 38% decrease in ED care costs, no differences in MACE
	BEACON trial [29] 2016	RCT	500 patients (CCTA vs. SOC) suspected NSTE-ACS in ED with normal high sensitivity cTn	N° patients identified with ob-CAD requiring PCI, direct discharge rate from the ED, length of hospital stay, costs, and rehospitalization for recurrent chest pain	No differences for all endpoint except for reduced outpatient testing and medical costs in CCTA group
	RAPID CTCA trial [30] 2021	RCT	1,748 patients (CCTA vs. SOC) with suspected NSTE-ACS in ED with at least one: ECG ischemic anomalies, history of ischemic heart disease, raised hs-cTn	Time to all cause death or non-fatal type 1 or type 4b AMI at one year	No difference for the prespecified endpoint but reduced ICA (with similar revascularization rates) and outpatient testing
	Puchner et al. [33] 2014	Observational	472 patients randomized to CCTA arm in the ROMICAT II trial	Rate of ACS during the index hospitalization	HRP significantly associated with ACS regardless of the degree of coronary stenosis and clinical predictors
	ICONIC study [35] 2018	Observational	234 ACS patients compared to 234 control pairs (non-ACS) underwent CCTA	Differences between the two groups regarding plaque features	Most culprit lesion of ACS cases were non ob-CAD. HRP had independent predictive value for ACS beyond clinical risk factors and plaque burden
	Kuneman et al. [37] 2023	Observational	66 ACS patients compared to 132 control pairs (stable CAD) underwent CCTA with PCAT attenuation analysis	Comparison of PCAT attenuation across precursors of culprit and nonculprit lesions within the two groups	The mean PCAT attenuation was significantly increased across culprit lesion in patients with ACS, compared to nonculprit lesions of these patients and to lesions of patients with stable CAD
	Linde et al. [60] 2020	Observational (from Verdict trial)	1,023 patients with suspected NSTE-ACS and at least one: ECG ischemic anomalies and raised hs-cTn (all performed CCTA and ICA)	Accuracy of CCTA to rule out/in coronary stenosis ≥50%, using ICA as the reference standard	CCTA showed a NPV of 90.9% and a PPV of 87.9% in patients with NSTE-ACS
	Kofoed et al. [61] 2021	Observational (from Verdict trial)	978 patients with suspected NSTE-ACS and at least one: ECG ischemic anomalies and raised hs-cTn (all performed CCTA and ICA)	CCTA vs. ICA for prognostic assessment in patients with NSTE-ACS (median follow up of 4.2 years)	CCTA was equivalent to ICA, with both methods MACEs were 1.7-fold higher in patients with ob-CAD than in those with non ob-CAD

The CT-STAT trial [28] demonstrated the superiority of a CCTA-based strategy over nuclear myocardial perfusion imaging (MPI) in this setting.

However, these previous studies did not use the hs-cTn assay, which increases sensitivity and specificity in the diagnosis of ACS. This omission may explain the divergent results of the BEACON trial [29] which used hs-cTn and showed that while CCTA as initial test was cost-effective, it was not superior to SOC in detecting significant CAD requiring PCI nor in reducing the length of hospital stay.

Importantly, these RCTs excluded patients with myocardial injury (abnormal cTn levels) emphasizing the low pre-test risk of ACS in the in the study populations.

According to the current ESC guidelines [1] in suspected ACS cases with non-elevated (or uncertain) hs-cTn, no ECG changes, and no recurrence of pain, incorporating CCTA or a non-invasive stress imaging test should be considered in the initial evaluation. Specifically, the recommendation class for the use of CCTA has shifted from I to IIa, following the Rapid CTCA trial [30] which provided data in higher risk patients **(**Fig. 2, case 1 and 4), and showed no reduction in all-cause mortality or non-fatal AMI at 1 year from the index event but reduced ICA, with similar revascularization rates. This suggests that CCTA may avoid unnecessary ICA without affecting coronary revascularization rate.

CCTA allows specific evaluation of plaque composition, quantification of total plaque volume and assessment of high-risk features such as positive remodelling, low-attenuation plaque, napkin-ring sign and spotty calcium [21] **(**Fig. 2, case 3).

In the context of acute chest pain and suspected NSTE-ACS, the updated CAD-RADS 2.0 classification system is used to assess the presence of CAD and plaque burden, to guide patient management decisions [31].

While standard use of CCTA as a first-line imaging investigation for suspected NSTE-ACS is not currently recommended, future research may change this recommendation [1].

### Prognostic Role

CCTA may provide important prognostic information in ACS patients. Indeed, recent findings from the PARADIGM study [32] and other studies suggest a possible application of CCTA for the follow-up of CAD progression in different clinical settings. High-risk plaques were more frequent in patients with suspected ACS and remained a significant predictor of ACS during the index hospitalization independent of degree of CAD and clinical risk assessment [33]. Notably, an atherosclerotic plaque with at least two high-risk characteristics has a particular high-risk of MACE [34].

In addition, as shown in the ICONIC study, although the risk of developing ACS increases with the severity of coronary stenosis, most precursors of culprit lesions in ACS are non-obstructive and therefore plaque assessment identifies high-risk patients above and beyond stenosis severity and aggregate plaque burden [35].

Pericoronary adipose tissue (PCAT) plays a role in the development and progression of coronary artery calcification and coronary plaque vulnerability [36]. Higher PCAT attenuation index values correlate with a higher risk of plaque rupture in NSTEMI patients. The mean PCAT attenuation index is significantly increased in culprit lesions in patients with ACS compared to tissue surrounding non-culprit lesions of these patients and lesions of patients with stable CAD. PCAT attenuation on CCTA may be a novel marker to identify high-risk plaques [37], as demonstrated in several other clinical settings [38].

Dysregulated PCAT has also been associated with AMI with non-obstructive coronary arteries (MINOCA) and coronary vasculitis. Assessment of PCAT can be useful to guide targeted primary prevention and ultimately to potentiate secondary prevention in patients at higher risk of MACE [39].

## Cardiac Magnetic Resonance

### Diagnostic Phase

The ESC guidelines for the management of ACS recommend CMR in patients with poor echocardiographic windows that limits a structural and motion assessment [1]. The use of CMR in the early phase of an ACS is more limited due to the reduced availability of CMR in ED, high cost, the duration of the examination, and the possible instability of these patients leading to motion and tracking artefacts. Because of these limitations, the evidence for the use of CMR in this setting remains limited.

However, CMR has shown high sensitivity in detecting early signs of ischemia. Cine imaging shows RWMA for several hours after transient ischemia due to myocardial stunning, and by using perfusion sequences, it is possible to assess ischemic areas with reduced myocardial blood flow (MBF) [40].

**Fig. 3:**
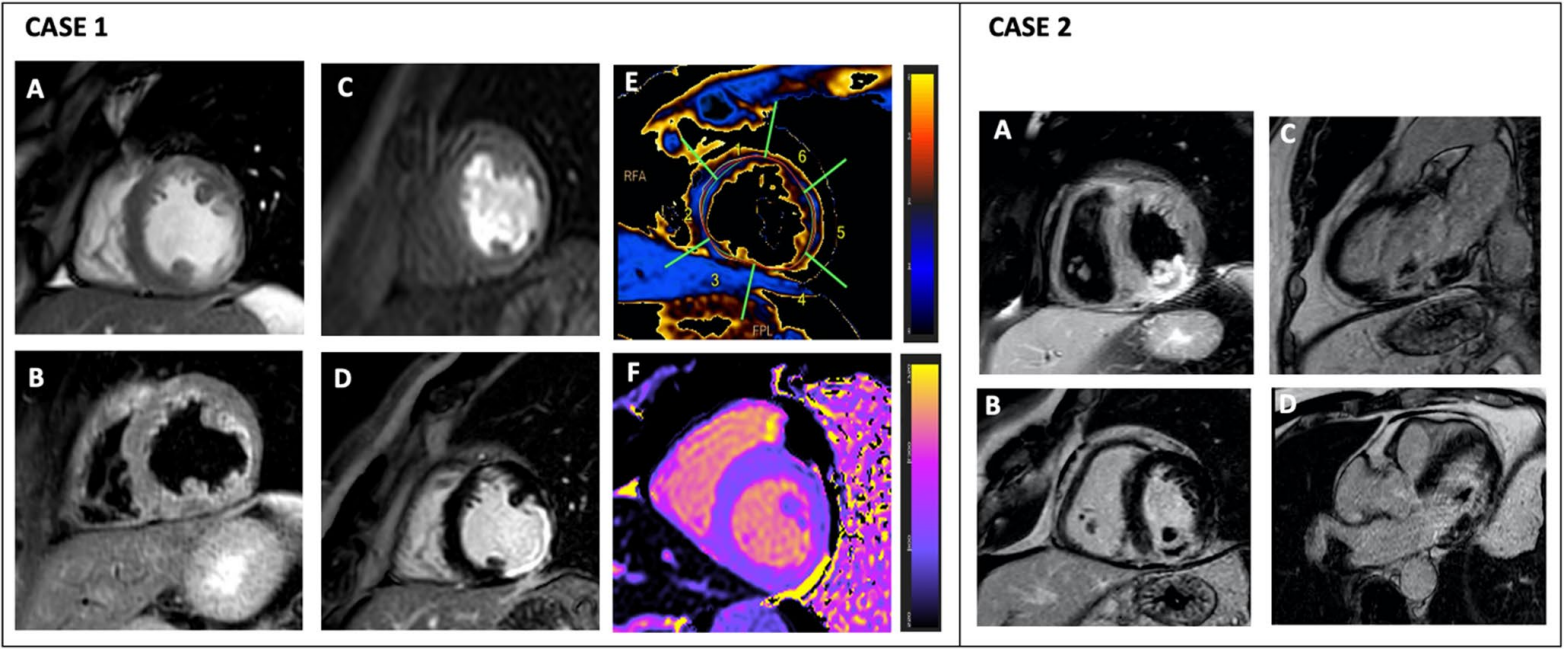
CMR evaluation in ACS.* Case 1*: CMR in a 38-year-old man post-AMI showed wall thinning in the mid-apical-lateral segment (1A), mild lateral wall edema on T2-weighted sequences (1B), perfusion deficit at rest (1C), and extensive subendocardial LGE (1D). Mapping revealed increased T2 (1E) and T1 (1F) relaxation times in the mid inferolateral segment. *Case 2:* CMR in a 75-year-old man after inferior STEMI showed edema in the mid-inferior segment, with hypointensity on T2 suggesting IMH (2A), transmural LGE confirming myocellular damage and ischemia affecting the postero-medial papillary muscle (2B, 2C, 2D). Abbreviations:AMI, acute myocardial infarction; STEMI, ST-elevation myocardial infarction; IMH, intramyocardial hemorrhage, and as inFigure 1

Identification of an ischemic pattern of late gadolinium enhancement (LGE) with concomitant edema (T2 STIR sequences or mapping) in patients with suspected ACS confirms the diagnosis, helps to establish the timing of AMI and to identify the culprit lesion [41] (Fig. 3, case 1). In addition, an imaging approach combining LGE and T2-weighted CMR accurately differentiates AMI from previous myocardial infarction [42]. However, in the very early phase, when the scar has not yet developed, LGE may overestimate the size of the infarction size because the abnormal tissue is enlarged by edema and partial volume effect due to inflammatory cells infiltration [43].

The myocardial salvage index (MSI) is obtained by combining area at risk (AAR) and infarct size (IS). It indicates how much of the myocardium at risk has been saved by timely revascularization and provides a measure of the effectiveness of acute interventions. Therefore, assessment of MSI is useful in clinical practice to better understand the myocardial area that can be salvaged after ischemic injury [44].

In addition, stress CMR may be useful in delineating ischemic areas. Currently stress CMR in ACS has shown a high diagnostic accuracy in identifying reversible myocardial perfusion deficits after gadolinium administration and inducible RWMA indicating flow-limiting coronary artery stenosis [45]. It can be used as an alternative to CCTA in the evaluation of patients in the observation zone following ECG and hs-cTn assessment, particularly in those with advanced and established CAD where insights into myocardial perfusion and viability may provide more useful information than CCTA [1].

Among the main findings **(**Table 3**)**, the CARMENTA trial compared CMR or CCTA-first strategy with routine clinical care in patients with suspected NSTEMI. ICA was recommended if the initial CMR or CCTA suggested a coronary etiology and was discouraged in the case of non-coronary etiology (e.g. myocarditis, PE). CMR and CCTA-first strategies led to a significant reduction in ICA (87% and 66%, respectively) compared with SOC, with no difference in MACE at one year [46].

**Table 3:** Selected studies on the diagnostic and prognostic role of CMR in the ACS setting. CMR, cardiac magnetic resonance; MINOCA, myocardial infarction with non-obstructive coronary arteries; cTnT, cardiac troponin T; IS, infarct size; MVO, microvascular obstruction; MSI, myocardial salvage index; IMH, intramyocardial hemorrhage; HF, heart failure; LGE, late gadolinium enhancement

IMAGING METHOD	STUDY	STUDY DESIGN	POPULATION	ENDPOINT	RESULTS
CMR	CARMENTA trial [46] 2018	RCT	207 patients with suspected NSTEMI (acute chest pain + raised hs cTnT) randomized at 3 strategies-first (CMR vs. CCTA vs. SOC)	Whether CMR or CCTA may serve as a safe gatekeeper for ICA (referral to ICA during hospitalization and 1-year outcomes	The CMR and CCTA-first strategies reduced ICA compared with SOC (87%, 66% and 100%, respectively), with similar outcomes
	CMR-IMPACT trial [47] 2023	RCT	312 patients (CMR vs. invasive strategy) with suspected NSTEMI (acute chest pain + mildly raised hs cTnT, max 1,000 ng/L)	Composite of death, AMI, and cardiac-related hospital readmission or ED access and secondary outcomes	No difference in clinical and safety event rates. CMR-based pathway facilitated safe discharge, increased the therapeutic yield of angiography (64,2% vs. 40%), and reduced ICA
	Shanmuganathan et al. [48] 2024	Trial	100 patients with suspected NSTEMI (acute chest pain + raised hs cTnI) underwent CMR before ICA	Diagnostic utility of CMR before ICA	CMR identified AMI in 67% of patients (84% in ob-CAD patients and 22% in non ob-CAD patients). Reclassified the presumed MINOCA in 67% of cases
	Mileva et al. [49] 2023	Meta-Analysis	3,624 patients with suspected MINOCA underwent CMR	Diagnostic and prognostic value of CMR in MINOCA patients	MINOCA confirmed in only 22% of cases and 68% of patients were reclassified after CMR in myocarditis, Takotsubo and normal findings
	TITAN-MRI trial ongoing	Trial	All patients with NSTEMI eligible for the study undergo CMR prior to ICA	Reclassification rate, culprit lesion identification, CMR’s effect on revascularization strategy	Ongoing
	Stone et al. [50] 2016	Meta-Analysis	IS assessed by CMR in 1,889 patients and by SPECT in 743 patients after pPCI (3–10 days) in STEMI	Prognostic value of IS	IS is strongly associated with all-cause mortality and hospitalization for HF within 1 year
	De Waha et al. [51] 2014	Trial	278 STEMI patients reperfused by pPCI underwent CMR 3 days after AMI	Prognostic value of IS, MVO, MSI and CMR-LVEF	MSI was independent predictor of MACE; model including CMR parameters on top of traditional outcome markers showed an incremental prognostic value
	Hamirani et al. [53] 2014	Meta-Analysis	2,435 patients underwent CMR after AMI	Prognostic value of MVO and IMH	MVO and IMH were associated with lower LVEF, increased LV volumes, IS and MACE; late MVO was a stronger outcomes predictor of MACE than early MVO
	Lechner et al. [54] 2024	Meta-Analysis	1,109 STEMI patients underwent CMR 3 days after pPCI	Prognostic value of different microvascular injury patterns	IMH was linked with larger IS, lower LVEF and was the only independent predictor of MACE. Patients with only MVO (MVO+/IMH-) had a similar outcome to patients without microvascular injury (MVO-/IMH-)
	Bergamaschi et al. [59] 2024	Observational	198 MINOCA patients underwent CMR during hospital stay	Prognostic value of CMR parameters	%LGE and abnormal T2 mapping values were independent predictors of MACE at 3 years of follow-up

Similarly, the CMR-IMPACT trial showed that a CMR-first approach facilitated safe discharge and reduced ICA over long-term follow-up [47].

A recent study showed that performing CMR prior to ICA in suspected NSTEMI patients, effectively discriminates AMI from non-ischemic pathologies. In fact, CMR confirmed AMI in 84% of patients with ob-CAD but only in 22% of patients without ob-CAD, reclassifying the NSTEMI diagnosis in 67% of cases [48]. Currently, CMR is essential in the differential diagnosis of acute myocardial damage without ob-CAD, identifying conditions such as MINOCA and myocarditis [49].

Ongoing research, including the TITAN-MRI study, aims to further clarify the diagnostic role of CMR in suspected NSTEMI prior to ICA.

### Prognostic Role

CMR plays a crucial role in the prognostic assessment of ACS, especially after STEMI. Key prognostic parameters include IS and AAR, which, together with the MSI, are predictive of outcomes in post-AMI patients. A patient-level analysis of ten RCTs showed that IS measured after pPCI is strongly associated with all-cause mortality and hospitalization for heart failure within one year [50].

In STEMI patients reperfused by pPCI, MSI is an independent predictor of MACE, with studies showing that models incorporating CMR parameters provide incremental prognostic value beyond traditional markers [51].

CMR also assesses myocardial reperfusion failure through metrics such as microvascular obstruction (MVO) and intramyocardial hemorrhage (IMH), both indicative of severe ischemic injury.

As demonstrated in a large meta-analysis MVO is associated with a lower LVEF, increased ventricular volumes and IS, and a higher risk of MACE. Late MVO is a better predictor of chronic heart failure, death, and recurrent AMI than early MVO. Furthermore, MVO extent ≥ 2.6% of LV improved long-term risk stratification over traditional outcome predictors [52]. IMH also correlates with adverse LV remodeling, reduced LVEF and MACE [53, 54] **(**Fig. 3, case 2). For this purpose, CMR score (combining CMR-LVEF, MSI, MVO, and IMH) was independently associated with MACE with the highest net reclassification improvement as compared to GRACE score and TTE-LVEF [55].

In addition, higher T1 and T2 values in non-infarcted myocardial areas after STEMI have been associated with adverse LV remodeling and worse cardiovascular outcomes [56, 57].

Recent evidence also suggests that hepatic T1 mapping values are associated with right ventricular dysfunction and incidence of heart failure after STEMI [58].

In MINOCA patients, early CMR findings, including %LGE and abnormal T2 mapping, independently predict MACE at three years and serve as high-risk markers [59].

## Diagnostic Pathway for Suspected NSTE-ACS: Role of non-invasive Imaging

Patients presenting with chest pain or its equivalent should receive a clinical history and physical examination, laboratory tests (including cTn levels), and prompt ECG execution. While a patient with suspected STEMI follows a well-defined and primarily interventional pathway, the management options for patients with suspected NSTE-ACS may vary (Graphical abstract). A baseline TTE to assess LVEF and RWMA is always performed and can be complemented by tools such as WMSI and STE, which can provide valuable information for risk stratification. In addition, in some specific cases, SE/stress MCE can help to evaluate myocardial ischemia and viability. If the echocardiographic findings suggest new ischemic changes, ICA would be indicated.

A routine invasive strategy carries a higher peri-procedural risk and an increased likelihood of bleeding, mainly due to the concomitant use of antithrombotic therapies. Therefore, in patients with suspected UA or NSTEMI but low levels of cTn, low pre-test probability of CAD, or possible alternative causes of myocardial injury, CCTA may be an excellent gatekeeper to rule out ob-CAD and thus avoid ICA.

As demonstrated in a sub-analysis of the VERDICT trial CCTA may be beneficial in patients with high clinical suspicion of NSTE-ACS to exclude CAD ≥ 50%. In these specific cases, there is no indication for revascularization, and the risk-benefit ratio of routine ICA may not be favorable [60].

Other evidence has shown that CCTA is equivalent to ICA for the assessment of long-term prognosis, in fact their findings were concordant in 88.5% of cases. Importantly, subsequent ICA in patients with non ob-CAD on CCTA or vice versa did not add further risk stratification [61].

Furthermore, the role of CMR is crucial in the differential diagnosis of acute myocardial injury with non-ob CAD after ICA (suspected MINOCA). Moreover, CMR and CCTA used as initial diagnostic tools seem to improve the selection of patients who would benefit from ICA, as shown in CARMENTA trial [46], suggesting that they may have an increasing diagnostic role in the future.

Nuclear imaging techniques, such as single photon emission tomography (SPECT) and positron emission tomography (PET) play an important role in the functional assessment of ischemia and myocardial viability in this setting, especially when CCTA cannot be performed, and in experienced centers they are performed in less than 30 min.

Finally, the prognostic role of non-invasive imaging modalities could directly aid the subsequent management of ACS patients. Table 4 provides a comprehensive review of the characteristics of use of the aforementioned non-invasive methods.

**Table 4:** Non-invasive imaging application overview in ACS setting. R/LVEF, right/left ventricular ejection fraction; RWMA, regional wall motion abnormalities; SE, stress echocardiography; STE, speckle-tracking echocardiography; GLS, global longitudinal strain; HCM, hypertrophic cardiomyopathy; UEA, ultrasound enhancing agents; FFR-CT, fractional flow reserve computed tomography; TRO, triple rule-out; PCAT, pericoronary adipose tissue; AAR, area at risk; IS, infarct size; MBF/MBFR, myocardial blood flow/reserve; MVO, microvascular obstruction; PCI, percutaneous coronary intervention; CABG, coronary-artery bypass graft; ICU, intensive care unit; ICD, implantable cardioverter defibrillator; late iodine enhancement. For others see previous abbreviations

5W FOR IMAGING IN ACS	WHAT FOR	WHO	WHEN	WINNING EDGE	WEAKNESS
TTE	-LVEF, RWMA with WMSI -STE (mainly GLS) -Exclusion of valvular heart diseases, type A aortic dissection, PE, HCM, and TTS, post-infartual mechanical complications. -Possible addition of UEAs and pharmacological stressors (stressMCE)	- All patients with suspected or confirmed ACS	-Diagnostic phase (first-line imaging modality) -Prognostic role (before discharge and during subsequent follow-up)	- Quick - Low-cost - Non-invasive and risk-free	-Operator-dependent -Acoustic window dependency -Expertise requirements
CCTA	- CAD anatomical evaluation (degree of stenosis, plaque burden, coronary anomaly) - CAD qualitative evaluation (HRP*) - Functional assessment (FFR-CT) -Rule out including concurrent (TRO) other causes of acute myocardial injury (PE, aortic dissection) -Additional information: PCAT attenuation index, Wall motion analysis, myocardial fibrosis evaluation (LIE)	-Patients at intermediate-low risk of NSTE-ACS - Avoid in unstable patients, very high risk NSTEMI, previous PCI/CABG †	-Diagnostic phase, at patient admission (ED or ICU) - Suspected extracardiac causes - CAD progression follow-up	-Rapid and non-invasive - High sensitivity and specificity, especially in low-risk patients -Exclusion of multiple causes of acute myocardial injury, even simultaneously (TRO) -High reproducibility -Early and safe discharge from the ED - Proven prognostic role associated	-Exposure to ionizing radiation -Risk of contrast-induced nephropathy -Not available at all centers -ECG gated and low heart rate (preferably < 70 bpm) required
CMR	- Function assessment (LVEF, RVEF and volume), RMWA - Tissue characterization: AAR, IS, MVO, IMH - Mechanical complications - Perfusion deficit at rest and after pharmacological stress	- Diagnostic work-up of suspected MINOCA - STEMI patients for prognostic assessment	- Diagnostic phase, 3–5 days after acute event - Prognostic phase, in patients with STEMI during hospitalization - Follow-up, 3–4 months after the acute event in high-risk patient or pre-ICD implantation	- High diagnostic accuracy - Non-invasive - Minimal risk - Multiparametric assessment - Tissue characterization	- High costs and long duration - Limited use in acute phases - Claustrophobia - Not feasible in patients with metallic devices (e.g., pacemaker), not available in many centers - Not usable in severe chronic renal disease
Nuclear imaging (SPECT, PET)	- Myocardial perfusion deficit at rest and after pharmacological/physical stress (it can replace SE) - Myocardial viability - Only PET: quantification of MBF and MBFR (useful to detection microvascular dysfunction, e.g. MINOCA)	- Patients at intermediate-low risk of NSTE-ACS	- Diagnostic phase, usually when CCTA not available	- High diagnostic accuracy - Non-invasive - Minimal risk	- Difficulty in distinguishing between acute and previous myocardial damage - High radiation exposure - Long duration (depending on local expertise) - Availability and cost (PET) - Balanced ischemia (only for SPECT)

* HRP (high-risk plaque), two of these features: napkin-ring sign, spotty calcium, positive remodeling, low-attenuation

† Even if new scan can detect restenosis in this setting

## Nuclear Imaging

European guidelines suggest that, depending on local expertise and availability, SPECT may be used in patients with non-elevated hs-cTn and normal ECG who could not undergo CCTA during the observation period or shortly after discharge [1]. Several studies **(**Table 5**)** have demonstrated that rest SPECT for patients with chest pain and an intermediate to low risk of ACS is associated with shorter length of stay in ED, lower costs and can reduce unnecessary hospitalizations showing an elevated NPV [62–68]. For example, Udelson et al. conducted a RCT among 2475 ED patients with chest pain or other symptoms suggestive of acute cardiac ischemia and with normal or nondiagnostic initial ECG, demonstrating that Tc-99 m sestamibi SPECT reduced unnecessary hospitalizations among patients without acute ischemia, without compromising appropriate admissions for those with acute ischemia. Although the high NPV, the role of resting SPECT is limited in distinguishing chronic from acute ischemia because a fixed perfusion defect may represent either an AMI or a chronic, non-viable, myocardial scar [69]. Stress MPI, with exercise or pharmacologic stress, may be used to detect the presence and extent of inducible perfusion abnormalities suggestive of ischemia, as well as the presence of prior infarction. Stress SPECT is comparable to CCTA in terms of diagnostic time, length of stay, and costs for low to intermediate risk patients in the ED [70]. While CCTA provides faster results, the use of tetrofosmin as a tracer can reduce acquisition time to 15 min without compromising image quality, effectively identifying severe myocardial ischemia and RWMA [71]. Moreover, stress TTE and MPI are both safe and diagnostic in acute chest pain patients who do not present recurrent ischemic symptoms, remain hemodynamically stable, with a negative initial evaluation with biomarkers and ECG [72]. When added to a standard triage strategy stress Tc-99 m tetrofosmin SPECT improved clinical decision making, significantly reducing the need for hospitalization without an increase in MACE rates at 30 days or 1 year [73]. Direct head-to-head studies, such as those by Forster et al. [74] and Kisacik et al. [75], show that dobutamine stress TTE and SPECT have comparable diagnostic accuracy for CAD, with SPECT demonstrating slightly higher sensitivity and SE slightly higher specificity, though differences are generally not statistically significant. Agreement between the two modalities is highest in patients without prior myocardial infarction. The choice between modalities is often guided by local expertise, patient characteristics, and test availability. PET imaging with tracers such as rubidium-82, N-13 ammonia, O-15 water, and F-18 flurpiridaz, improves the detection of perfusion abnormalities, allows the assessment of LV function at rest and during stress, and it can measures MBF and MBF reserve [76]. PET offers several advantages over SPECT, including improved diagnostic accuracy, lower radiation exposure, fewer equivocal studies especially in case of balanced ischemia, and shorter ED stays [77]; furthermore, PET is particularly advantageous in patients with multivessel disease or high body mass index, where SPECT and TTE may be less reliable. By quantifying MBF reserve, PET assists in the diagnosis of microvascular angina and epicardial CAD and provides prognostic information. ^13^N-ammonia PET with normal MBF reserve correlates with a three-year safety period with a low risks of MACE [78]. Although nuclear imaging can be time-consuming, it can provide important information, such as the association of ^18^F-NaF uptake with high-risk plaque characteristics in ACS patients [79].

**Table 5:** Selected studies on the diagnostic role of nuclear imaging in the ACS setting

IMAGING METHOD	STUDY	STUDY DESIGN	POPULATION	ENDPOINT	RESULTS
Nuclear Imaging (SPECT, PET)	Udelson et al. [62] 2002	RCT	2475 ED patients (SOC only vs. SOC + rest Tc-99 m sestamibi SPECT) with chest pain or other symptoms suggestive of acute ischemia with normal/nondiagnostic ECG	Whether incorporating rest SPECT improves clinical decision making for initial ED triage	SPECT reduced unnecessary hospitalizations by 10% among patients without acute ischemia, without reducing appropriate admission for patients with acute ischemia.
	Nabi et al. [70] 2016	Observational	598 ED low-to-intermediate risk patients with chest pain (CCTA vs. stress only SPECT)	Length of hospital stay, other endpoints were test feasibility, time to diagnosis, diagnostic accuracy, radiation exposure, and overall cost	Stress SPECT when optimized with stress-only imaging is similar to CCTA in time to diagnosis, LOS, and cost, with improved prognostic accuracy and less radiation exposure
	Lim et al. [73] 2013	RCT	1,508 ED patients with chest pain with nondiagnostic ECG and negative cTn (randomized 2:1 for SOC + stress Tc-99 m tetrofosmin SPECT vs. SOC only)	MACE at 30 days or 1 year follow up and hospitalization during the index event	Stress SPECT reduced admission rate than SOC only (10.16% vs. 18.45%), with no significant between-group differences in MACE after 30 days or 1 year
	Majeed et al. [79] 2021	Observational	62 ACS patients underwent multi-vessel OCT, 18 F–NaF PET and CCTA	Diagnostic value of 18 F–NaF PET for plaque characterization	18 F–NaF uptake is associated with high-risk plaque features on OCT and CTCA in a per-segment and per-patient analysis in subjects hospitalized for ACS.
	Conti et al. [65] 2001	Observational	231 ED patients with chest pain within 24 h from onset (negative ECG, cTn and TTE) underwent rest Tc-99 m sestamibi/tetrofosmin SPECT (if < 3 h from onset) or exercise SPECT (if ≥ 3 h)	Detection of significant CAD by ICA and MACE at 6 months	High NPV (99%) and same accuracy between rest and stress SPECT in these two population
	Hilton et al. [66] 1996	Observational	150 ED patients with typical chest pain and normal/nondiagnostic ECG underwent rest Tc-99 m sestamibi SPECT	MACE at 90-days follow up	Among 87 patients with a normal SPECT there were no MACE at 90 days vs. 8% of MACE in patients with abnormal SPECT
	Schaeffer et al. [67] 2007	Observational	479 ED patients with chest pain and nondiagnostic ECG underwent rest Tc-99 m tetrofosmin SPECT	Utility of rest SPECT, including an overnight delayed image acquisition protocol, at 30-days follow up	A normal rest SPECT predicts a very low occurrence of MACE (99.3% of NPV). A delayed image acquisition protocol did not decrease the accuracy
	Radensky et al. [68] 1997	Observational	102 ED patients with chest pain and nondiagnostic ECG underwent rest Tc-99 m sestamibi SPECT vs. 107 patients with SOC strategy	Cost-effectiveness of SPECT	Mean costs per patient of the SPECT strategy and NO SPECT strategy were $5,019 versus $6,051, respectively.

Technetium-SPECT can assess IS after pPCI, as predictive marker for MACE [50], but it is unable to analyze other parameters like microvascular obstruction or intramyocardial hemorrhage.

In conclusion, rest/stress SPECT and PET, although not first choice in ACS setting, are valuable in the assessment of chest pain when CCTA is not suitable or inconclusive, particularly for quantifying myocardial ischemia and assessing viability.

## Conclusions and Future Perspectives

Multimodal non-invasive imaging is essential for the comprehensive evaluation of patients with ACS providing both diagnostic and prognostic insights. Given the costs and risks associated with invasive procedures such as ICA, non-invasive approaches are critical in this context. Several factors need to be considered, including clinical presentation, likelihood of ob-CAD, and potential high-risk features that may necessitate urgent revascularization over a non-invasive strategy. The use of non-invasive imaging may be particularly beneficial in cases of non-high-risk NSTEMI or suspected alternative diagnoses, including suspected MINOCA. These imaging modalities should be tailored to individual patient characteristics and available resources, with the aim of reserving ICA for high-risk cases.

Dedicated trials are needed to further elucidate the safety and efficacy of non-invasive approaches in ACS to ensure that patients receive the most appropriate care while minimizing unnecessary risks associated with invasive procedures.

## Key References

- **Rapid CTCA Trial.** Gray AJ, Roobottom C, Smith JE, Goodacre S, Oatey K, O’Brien R, et al. Early computed tomography coronary angiography in patients with suspected acute coronary syndrome: randomised controlled trial. BMJ. 2021 Sep 29;374:n2106.
  - Findings from this study suggest that CCTA-first approach vs. standard of care in suspected NSTE-ACS reduced ICA (with similar revascularization rates) and outpatient testing.
- **CMR-Impact trial.** Miller CD, Mahler SA, Snavely AC, Raman SV, Caterino JM, Clark CL, et al. Cardiac Magnetic Resonance Imaging Versus Invasive-Based Strategies in Patients With Chest Pain and Detectable to Mildly Elevated Serum Troponin: A Randomized Clinical Trial. Circ Cardiovasc Imaging. 2023 Jun;16 (6):e015063.
  - Findings from this study suggest that CMR-first approach vs ICA in case of suspected NSTEMI patients facilitated safe discharge and reduced ICA over long-term follow-up
- Shanmuganathan M, Nikolaidou C, Burrage MK, Borlotti A, Kotronias R, Scarsini R, et al. Cardiovascular Magnetic Resonance Before Invasive Coronary Angiography in Suspected Non-ST-Segment Elevation Myocardial Infarction. JACC Cardiovasc Imaging. 2024 Sep;17(9):1044-1058.
  - Findings from this study suggest that CMR before ICA has the potential to change diagnosis and/or management in at least 50% of patients presenting with presumed “Acute NSTEMI”.

## Data Availability

No datasets were generated or analysed during the current study.

## Abbreviations

AAR: area at risk
ACS: acute coronary syndrome
AMI: acute myocardial infarction
CAD: coronary artery disease
CCTA: coronary computed tomography angiography
CMR: cardiac magnetic resonance
CT: computed tomography
cTn: cardiac troponin
ECG: electrocardiogram
ED: emergency department
GLS: global longitudinal strain
hs-cTn: high-sensitivity cardiac troponin
ICA: invasive coronary angiography
IMH: intramyocardial hemorrhage
IS: infarct size
LGE: late gadolinium enhancement
LV: left ventricular
LVEF: left ventricular ejection fraction
MACE: major adverse cardiovascular events
MBF: myocardial blood flow
MCE: myocardial contrast echocardiography
MINOCA: myocardial infarction with nonobstructive coronary arteries
MPI: myocardial perfusion imaging
MSI: myocardial salvage index
MVO: microvascular obstruction
NPV: negative predictive value
NSTE-ACS: non-ST-elevation acute coronary syndrome
NSTEMI: non-ST-elevation myocardial infarction
Ob-CAD: obstructive CAD
PCAT: pericoronary adipose tissue
PCI: percutaneous coronary intervention
pPCI: primary percutaneous coronary intervention
PE: pulmonary embolism
PET: positron emission tomography
RCT: randomized controlled trial
RWMA: regional wall motion abnormalities
SE: stress echocardiography
SOC: standard of care
SPECT: single photon emission tomography
STEMI: ST-elevation myocardial infarction
TTE: transthoracic echocardiography
UA: unstable angina
WMSI: wall motion score index
